# The Victimisation of V.A.D.s.

**Published:** 1919-10-04

**Authors:** 


### The Victimisation of V.A.D.s.

*To the Editor of* The Hospital.

Sir,—May I presume to send a few lines in sympathy with J. E. Green, whose letters appeared in The Hospital last week *re* V.A.D. gratuity? Perhaps she is not aware that apparently a very small percentage of V.A.D. nurses are to get it, as I am told, on applying, that only those nurses who have worked in military hospitals are entitled to it. I have done three years' nursing in a first-line military auxiliary hospital of over two hundred beds, where we were under signed contracts every six months, and we got none of the advantages that a V.A.D. did in military hospital or uniform allowance, and then when an announcement is made that we are to receive a gratuity, and we apply for it, we are calmly told we are not entitled to it. It is, I think, a great injustice, and one that should be made public. We did not ask for a gratuity, or expect any such thing; but I do think, in general fair play, if one is to get it, all should; also, the general service members who served whole time, as the nursing staff did.—I am, yours truly,

